# Multifactor analysis of urban pluvial flooding using a comprehensive vulnerability index

**DOI:** 10.4102/jamba.v17i1.1835

**Published:** 2025-06-18

**Authors:** Eka Mutia, Azmeri Azmeri, Alfiansyah Yulianur, Ashfa Achmad, Ella Meilianda

**Affiliations:** 1 School of Engineering, Universitas Syiah Kuala, Banda Aceh, Indonesia; 2 Department of Civil Engineering, Faculty of Science and Technology, Samudra University, Langsa, Indonesia; 3 Department of Civil Engineering, Faculty of Engineering, Universitas Syiah Kuala, Banda Aceh, Indonesia; 4 Department of Architecture and Planning, Faculty of Engineering, Universitas Syiah Kuala, Banda Aceh, Indonesia

**Keywords:** pluvial flooding, urban vulnerability, Flood Vulnerability Index (FVI), drainage infrastructure, land-use planning, adaptive capacity, green space, flood mitigation

## Abstract

Urbanity is facing a growing threat of pluvial flooding due to rampant urbanisation and transformations combined with insufficient drainage systems. This situation becomes aggravated by flat topography and heavy rainfall, especially in poorly drained areas. Assessing flood vulnerability at the community level using the Flood Vulnerability Index (FVI) is essential for effective urban planning and flood risk management. The FVI consists of four key components: physical, environmental, social and economic. Physical factors encompass topography, rainfall and drainage systems, while environmental factors involve green spaces and retention zones. Social factors pertain to population density and community awareness, whereas economic factors relate to potential revenue losses and recovery capacity. The FVI was calculated using both secondary data and field observations, based on exposure, sensitivity and adaptive capacity. High population density, low green area ratio and poor drainage conditions are associated with high FVI, while when accompanied by higher environmental support and adaptive capacity in the community, lower vulnerability was observed.

**Contribution:**

This study highlights the comprehensive application of the FVI in urban flood risk management. The findings emphasise the need for systematic improvements in drainage infrastructure, sustainable management of natural resources and strategic land-use planning to enhance flood risk reduction. These insights provide a valuable contribution to the advancement of flood risk assessment and management frameworks, supporting more resilient urban planning strategies.

## Introduction

Floods are among the most prevalent natural disasters globally, affecting public health, the economy and infrastructure, with significant frequency and impact (CRED, UCLouvain & USAID 2023). According to the Emergency Events Database (EM-DAT), floods accounted for 41.10% of global disaster events in 2023, marking an 8.54% increase compared to 2022. However, despite these increases, the number of affected individuals decreased by 16.67%, indicating improvements in flood mitigation systems in many countries (CRED et al. 2023). Flooding in urban areas of Indonesia is worsened by unplanned urbanisation and climate change, which increases rainfall intensity. Typically, rapid urbanisation, land-use changes and insufficient drainage systems contribute to urban flooding (BNPB 2024).

The strain on urban drainage systems in large cities often results in an inability to handle significant rainwater volumes. Clogging from commercial waste further exacerbates urban drainage issues by obstructing water flow in drainage channels. Urban areas, particularly low-lying and flat regions, are especially prone to inundation. These low-lying zones often suffer disproportionately as water cannot drain effectively (Borbor-Cordova et al. 2020). Urban flooding severely disrupts daily activities, damages infrastructure and incurs economic losses. The absence of green spaces that could act as rain catchments worsens the situation, and settlements in flood-prone zones often lack flood-resistant infrastructure (Borbor-Cordova et al. 2020).

Flooding disproportionately impacts communities in crowded settlements with limited livelihoods, education access and disaster mitigation capacities. Those in lower socioeconomic tiers often reside in more vulnerable areas, such as riverbanks or lowlands, and face greater challenges in recovery after a disaster (Tate et al. 2021). Therefore, addressing urban flooding requires an integrated approach that includes infrastructure improvements, land-use policies and enhancing community resilience (Kittikhun et al. 2020). Flood risk assessments, incorporating exposure, sensitivity and adaptive capacity, are crucial for understanding urban vulnerability and developing effective mitigation strategies (Thorne et al. 2018; Borbor-Cordova et al. 2020).

Langsa city, located in Aceh Province, Indonesia, is highly susceptible to pluvial flooding. Its lowland topography, large rivers and significant annual rainfall (1850 mm – 4013 mm) contribute to this vulnerability (BPS Kota Langsa 2024). Rapid urbanisation, land-use changes and inadequate drainage infrastructure worsen the situation (Aksa & Afrian 2022). Various studies have suggested general flood mitigation strategies, such as strengthening drainage systems, spatial planning and land conservation (Borbor-Cordova et al. 2020; Kittikhun et al. 2020). However, Langsa’s unique geographical and socioeconomic attributes necessitate tailored solutions. The Flood Vulnerability Index (FVI) offers an approach that combines physical, social and economic parameters to evaluate flood vulnerability (Balica n.d.; Balica, Wright & Van der Meulen 2012).

This research aims to evaluate the flood vulnerability of Langsa using the FVI, which comprises four main components: physical, environmental, social and economic. The findings are critical for developing integrated recommendations for flood risk reduction, including infrastructure upgrades, land-use management and community preparedness. These measures are informed by the key contributing factors identified within each FVI component.

## Research methods and design

### Materials

**FIGURE 1:**
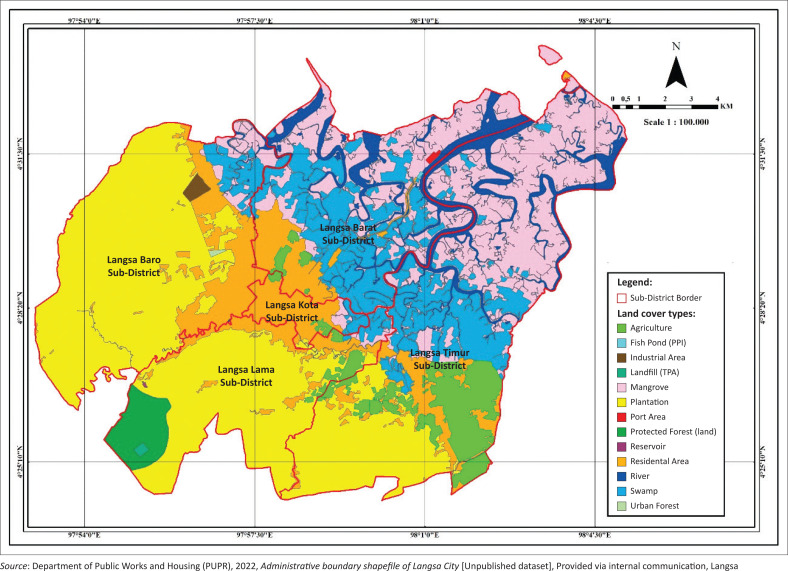
Langsa city map.

This study focused on Langsa city, Aceh Province (see Figure 1). Topographic maps, rainfall data and land use maps were used to support the hydrological analysis requirements. Demographic and socioeconomic profiles were also collected from the Langsa city Statistics Bureau (BPS Kota Langsa) to account for population density and other social determinants. Spatial analysis employed Geographic Information System (GIS) with stratified sampling in flood-affected areas. Field surveys and non-structured interviews were conducted with approximately 10–15 residents per subdistrict, as well as with neighbourhood and hamlet heads, to gather qualitative information on flood points, including water depth, inundation extent and recurrence. These interviews were aimed at verifying GIS findings and are not intended to be statistically representative, but rather to ground-truth spatial and observational data.

### Method

Various hydrological, spatial and statistical analyses were utilised to assess flood vulnerability in this study. Stratified sampling was conducted across five districts in Langsa city, incorporating rainfall patterns, population density and land use types. Spatial analysis was performed using ArcGIS to identify flood-prone areas, which were then validated through field surveys. The spatial data included land cover classification (e.g. forested areas, urban zones and water bodies), and the runoff coefficient was estimated using the Modified Rational Formula to assess surface water flow behaviour.

Flood vulnerability indicators were identified and grouped into four main components: physical, environmental, social and economic. Each indicator was selected based on its potential to either mitigate or exacerbate flood risk and was supported by literature and empirical observation. Physical indicators included rainfall intensity, topography and proximity to rivers or drainage systems. Environmental indicators encompassed land use, green space availability and infrastructure quality. Social factors involved population density, education level and access to sanitation, while economic indicators reflected investment in flood mitigation and spatial planning (Balica n.d.; Balica et al. 2012; Borbor-Cordova et al. 2020).

To evaluate the overall flood vulnerability level of each subdistrict, the FVI (Balica et al. 2012). The FVI incorporates three core dimensions: exposure, sensitivity and adaptive capacity, which were each measured using multiple indicators. Qualitative and quantitative data from field observations and secondary sources were standardised and converted into a scoring scale ranging from 1 (very low) to 5 (very high). The FVI score for each subdistrict was computed using the formula (Balica et al. 2012):

$$FVI=\frac{\left(Exposure+Sensitivity\right)}{Adaptive\;capacity}$$ [Eqn 1]

The FVI results were then classified into four categories:

- very high vulnerability: FVI > 0.75
- high vulnerability: 0.50 – 0.75
- medium vulnerability: 0.25 – 0.49
- low vulnerability: < 0.25

To interpret the classification and ensure consistency with field conditions, a rule-based framework was used. This framework integrates multiple criteria:

- Very High Vulnerability: Subdistricts with flat topography, high rainfall intensity (> 100 mm/day), poor drainage infrastructure and very high population density (above 6000 people/km^2^), coupled with limited sanitation access and weak adaptive capacity (e.g. low investment in flood mitigation).
- High Vulnerability: Areas meeting at least two major risk conditions, including population density between 800 and 6000 people/km^2^, inadequate drainage systems, limited evacuation infrastructure and inconsistent institutional support.
- Medium Vulnerability: Subdistricts with moderate topography, partial drainage coverage, population density between 500–799 people/km^2^ and moderate adaptive capacity.
- Low Vulnerability: Areas with favourable natural conditions (e.g. sloped terrain, vegetated land use), reliable infrastructure and low population density (below 500 people/km^2^), alongside consistent investment in flood control and sanitation services.

These classifications were validated through ground checks, stakeholder interviews and triangulation with GIS-based spatial data. The combination of standardised scoring, empirical observation and rule-based classification enables a robust and context-sensitive vulnerability assessment.

### Ethical considerations

Ethical waiver to conduct this study was obtained from the Universitas Samudra dated 03 October 2024 (No. 38.a/ UN54.6/PG/2024).

## Results

### Physical components

This study found that physical parameters, including rainfall, topography, proximity to rivers or estuaries and road and drainage systems, significantly impact flood vulnerability in Langsa city. Similar trends are observed in other coastal cities such as Bangkok, Jakarta and Miami, where these factors amplify flood risks and vulnerability, as also reported in similar studies of coastal cities (Yulianur et al. 2013; Zhu et al. 2021). These data indicate that physical factors dominate flood vulnerability in Langsa city, mirroring patterns seen in other cities globally (Balica et al. 2012; Mutia et al. 2021a).

#### Rainfall and topography

**FIGURE 2:**
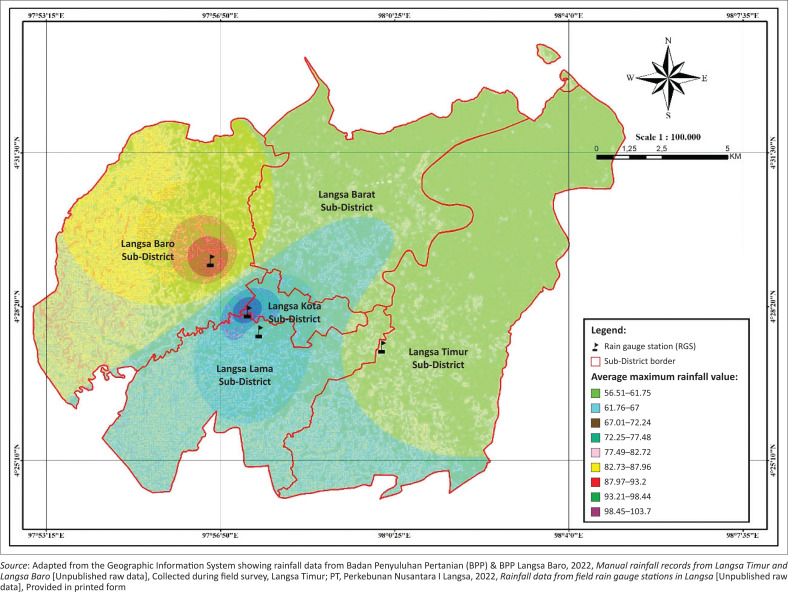
Langsa city map showing maximum rainfall distribution and topography in Langsa city.

Rainfall in Langsa city varies significantly across subdistricts, with Langsa Baro recording the highest maximum daily rainfall at 103.7 mm/day. While topography does not directly influence rainfall, it plays a crucial role in the distribution and management of rainwater. Approximately 66% of Langsa city consists of flat terrain with slopes ranging from 0% to 15%. This condition leads to water stagnation on these flat surfaces rather than rapid drainage, elevating the risk of ponding and flooding, especially in areas with inadequate drainage (Figure 2).

The flat topography slows water flow from areas such as Langsa Kota Subdistrict and Langsa Lama Subdistrict, resulting in water accumulation during heavy rainfall events. During extreme rainfall, these areas experience water stagnation due to inadequate drainage routes into low-lying zones or out of residential areas. In contrast, subdistricts with steeper topography allow for quicker water drainage towards lower areas, reducing surface water retention (Yulianur et al. 2013). Consequently, areas like Langsa Baro Subdistrict and Langsa Lama Subdistrict are particularly susceptible to flooding due to high precipitation (Romali & Yusop 2021; Zevri 2021).

#### Distance to rivers or estuaries

Proximity to rivers or estuaries significantly affects water flow and flood vulnerability in Langsa. For instance, drainage channels in areas like Sudirman Road have a low slope value (0.03), indicating a high potential for flooding. Similar challenges are seen in cities like Jakarta and New Orleans, where low-gradient channels impede efficient water flow, exacerbating flooding (Wesli & Hasyim 2013; Yulianur et al. 2013; Zevri 2021). The slow water flow in these low-slope channels increases the local flood risk during extreme rainfall events (Zhu et al. 2021).

#### Road and drainage infrastructure

Inadequate drainage systems in flat areas with 0% – 15% slopes, such as Langsa Kota Subdistrict and Langsa Lama Subdistrict, make flooding a prevalent risk during extreme rainfall. Due to the flat terrain, water flow is slower, leading to prolonged surface water retention compared to areas with better drainage (Yulianur et al. 2013; Zevri 2021). In contrast, subdistricts like Langsa Timur Subdistrict and Langsa Barat Subdistrict, which have better drainage systems, experience lower flood depths despite high rainfall, demonstrating the effectiveness of proper drainage infrastructure in reducing flood risk. Regions in Langsa with high rainfall, flat terrain and poor drainage are particularly vulnerable to flooding, emphasising the need for future infrastructure improvements to mitigate these risks (Table 1). Comparable situations occur in Johan Pahlawan Subdistrict, West Aceh Regency, where poor drainage leads to flooding (Azmeri & Satria 2021).

**TABLE 1:** Rainfall and flood depth data in five Subdistricts of Langsa city.

Subdistrict	Maximum daily rainfall (mm)	Flood depth (cm)	Drainage infrastructure
Langsa Kota	103.70	150	Inadequate
Langsa Barat	87.96	28	Adequate
Langsa Baro	103.70	60	Sufficient
Langsa Lama	103.70	56	Inadequate
Langsa Timur	82.72	20	Sufficient

*Source:* Rainfall data from Badan Penyuluhan Pertanian (BPP) & BPP Langsa Baro, 2022, *Manual rainfall records from Langsa Timur and Langsa Baro* [Unpublished raw data], Collected during field survey, Langsa Timur; PT, Perkebunan Nusantara I Langsa, 2022, *Rainfall data from field rain gauge stations in Langsa* [Unpublished raw data], Provided in printed form; flood depth and drainage infrastructure data from field surveys

Our analysis of road damage data indicates that many subdistricts show significant levels of severe damage. This widespread road damage is likely caused by intense rainfall combined with flat terrain, which leads to water ponding on the roads. Heavy rainfall, compounded by inadequate drainage in many subdistricts, causes prolonged water stagnation that quickly damages already fragile infrastructure (Tate et al. 2021). Langsa city should enhance its drainage system and employ risk mapping models to identify flood-prone areas for prioritised management (Borbor-Cordova et al. 2020), as floods not only threaten community safety but also damage infrastructure. Community-based mitigation strategies, including green technologies like rain gardens and permeable pavements, effectively reduce surface runoff and enhance community resilience to flooding. This approach underscores the importance of community involvement in applying sustainable, collaborative solutions for disaster risk mitigation and urban ecosystem preservation. These measures would strengthen Langsa city’s future preparedness for extreme rainfall events.

## Discussion

### Environmental components

Environmental factors, including land use, natural reserves and infrastructure quality, play significant roles in influencing flood vulnerability in Langsa city. Each component’s role in amplifying or mitigating flood impacts is crucial, particularly in rapidly growing urban areas (Bigi et al. 2021; Borbor-Cordova et al. 2020; Mutia, Lydia & Firdasari 2021b). Comparative studies with other global cities facing similar environmental challenges, such as Bangkok and Dhaka, show that poor environmental management exacerbates flood risks (Bigi et al. 2021).

#### Land use and natural reserves in flood mitigation

In Langsa city, land use changes involving urban expansion into green areas have increased flood vulnerability, similar to trends seen in Jakarta. Plantations and mangrove forests in subdistricts like Langsa Baro Subdistrict and Langsa Timur Subdistrict act as natural sponges, absorbing rainwater and reducing runoff. In highly urbanised areas like Langsa Kota Subdistrict, green spaces are less effective, resulting in higher flow coefficients, limited absorption and greater surface runoff, thus elevating flood risk. In contrast, natural areas like those in Langsa Timur Subdistrict, with lower flow coefficients, can absorb rainfall more effectively, whereas urbanised zones like Langsa Kota Subdistrict and Langsa Baro Subdistrict face higher flood risk due to higher flow coefficients.

To mitigate future flood risks, Langsa needs enhanced spatial planning, improved drainage systems, investment in protected water catchment areas and mangrove rehabilitation, particularly in high-risk areas like Lake city of Langsa and Langsa Baro Subdistrict (Zevri 2021).

#### Infrastructure quality

The quality of environmental infrastructure, such as drainage systems and roads, plays a critical role in Langsa’s capacity to manage heavy rainfall. Inadequate or nonexistent drainage systems, particularly in Langsa Kota Subdistrict and Langsa Timur Subdistrict, hinder water flow and heighten flood risks.

Modelling results indicate that poor infrastructure can prolong flooding by up to 30% compared to areas with more robust infrastructure. Urban areas like Manila and Lagos, with subpar infrastructure such as poor-quality roads and drainage, face higher flood susceptibility (Tate et al. 2021). The Langsa city government should enhance the protection of natural reserves, particularly in Langsa Timur Subdistrict and Langsa Baro Subdistrict. Reforestation of mangroves and the improvement of drainage infrastructure are essential for reducing flood susceptibility. Modelling results and global studies support that these measures will help reduce flooding and increase the city’s resilience to future flood events (Borbor-Cordova et al. 2020; Mutia et al. 2021).

### Social components

Social vulnerability in Langsa city can be assessed using indicators such as population density, education, access to clean sanitation and poverty levels. These factors affect the community’s capacity to prepare for, respond to and recover from flooding.

#### Poverty, population density and growth in Langsa city

For instance, Langsa city’s population density rose from 598 people/km^2^ in 2013 to 812 people/km^2^ in 2023, with Langsa Baro experiencing the highest growth rate among subdistricts at 2.03% (BPS Kota Langsa 2024). Increased population leads to greater pressure on infrastructure, especially drainage systems and evacuation spaces, thereby increasing flood risk. Additionally, lower educational attainment among the 16–18 age group in Langsa contributes to poor disaster preparedness (BPS Kota Langsa 2024). Addressing these vulnerabilities requires effective spatial planning and improved disaster mitigation education (Tate et al. 2021).

#### Access to education and sanitation

Limited access to sanitation exacerbates flooding by clogging drainage systems with sewage, while poor conditions perpetuate poverty and hinder community rehabilitation as well as recovery. While the poverty rate in Langsa declined from 12.08% in 2014 to 10.53% in 2023, gaps in infrastructure persist. Flood risks increase due to inadequate sanitation (Ajtai et al. 2023; Azmeri & Satria 2021) and insufficient disaster education (Mutia et al. 2021a; Tate et al. 2021). Community-based education programmes, like those conducted in Tokyo and Singapore, can empower vulnerable populations – such as low-income residents, individuals with limited access to sanitation and those living in low-lying flood-prone areas – and improve disaster preparedness. These programmes can significantly enhance resilience and recovery in Langsa, particularly among these at-risk groups (Azmeri & Satria 2021; Borbor-Cordova et al. 2020).

### Economic components

Economic aspects of flood vulnerability in Langsa city are shaped by urban planning and budget allocations for flood control infrastructure. These factors determine how the community and government can respond to and manage the effects of flooding (Borbor-Cordova et al. 2020).

Langsa city has implemented a Regional Spatial Planning policy for land use, but weak oversight has led to settlements being built in flood-prone areas like Langsa Kota subdistrict. Similar issues have increased flood risk in cities such as New Orleans (CRED et al. 2023).

Investment in flood control in Langsa City is unevenly distributed, with most funding directed to Langsa Baro, indicating prioritisation of flood reduction in this area. In 2023, Langsa Baro subdistrict received the highest allocation at 1616 billion IDR, followed by Langsa Barat subdistrict and Langsa Lama subdistrict. High-risk areas like Langsa Kota, which started receiving funding in 2022, require continued investment to minimise flood damage. Examples from cities like Miami and Singapore demonstrate that systematic infrastructure investment reduces economic exposure to flooding (Borbor-Cordova et al. 2020). Continuous funding allocations are necessary to address rising sea levels and prepare flood-prone areas in Langsa for future flood threats.

The analysis ranked flood vulnerability by subdistrict as follows: Langsa Kota (very vulnerable), Langsa Baro (vulnerable), Langsa Barat (moderate), Langsa Lama (vulnerable) and Langsa Timur (less vulnerable), based on land use and conservation area data (Table 2).

**TABLE 2:** Flood vulnerability levels in Langsa City.

Subdistrict	Physical and environmental	Social and economic	Vulnerability
Langsa Baro	Moderate drainage, flat topography, extensive green spaces	High population density, poor sanitation access, minimal evacuation routes, consistent investment in flood mitigation	High
Langsa Barat	Adequate drainage, varied topography, limited mangrove coverage	Medium population density, adequate residential conditions, improved sanitation, declining investment	Medium
Langsa Kota	Poor drainage, flat topography, minimal green spaces	High population density, inadequate sanitation access, fluctuating investment, economic centre	Very high
Langsa Lama	Inadequate drainage, mixed topography, limited green spaces	Medium population density, insufficient evacuation routes, low investment in flood control	High
Langsa Timur	Good drainage, predominantly agricultural land, extensive green spaces	Low population density, good sanitation, stable economic activities, consistent investment	Low

### Flood Vulnerability Index

The flood vulnerability analysis in Langsa city, performed using the FVI, evaluated exposure, sensitivity and adaptive capacity across subdistricts, considering physical, environmental, social and economic factors. Langsa Kota subdistrict exhibited a very high vulnerability level. Langsa Lama subdistrict and Langsa Baro subdistricts were found to have high vulnerability levels due to elevated flood exposure and sensitivity, compounded by limited adaptive capacity. In contrast, Langsa Timur subdistrict and Langsa Lama subdistricts demonstrated lower vulnerability levels, supported by stronger adaptive capacity and natural environmental benefits (Table 3). The key indicators affecting flood vulnerability, including physical, environmental, social, and economic factors contributing to the variation in vulnerability among subdistricts, are detailed in Table 4.

**TABLE 3:** Flood vulnerability level based on Flood Vulnerability Index.

Subdistrict	Exposure	Sensitivity	Adaptability	Vulnerability level
Langsa Baro	High	High	High	High
Langsa Barat	Medium	Medium	Medium	Medium
Langsa Kota	High	High	Low	Very high
Langsa Lama	High	Medium	Medium	High
Langsa Timur	Low	Low	High	Low

**TABLE 4:** Key indicators affecting flood vulnerability in Langsa city.

Indicator	Component type	Explanation
**Rainfall and topography** (Ajin et al. 2013)	Physical	Rainfall and topography impact water flow and flood risk.
**Proximity to rivers or drainage infrastructure** (Borbor-Cordova et al. 2020; Yulianur, Mutia and Sugianto 2013)	Physical	Location near rivers and drainage quality affect flood mitigation.
**Land use and natural reserves** (Romali and Yusop 2021; Zhu et al. 2021)	Environmental	Land use and presence of natural buffers like forests reduce flood impacts.
**Quality of infrastructure** (Sekar Ningrum and Br Ginting 2020)	Environmental	Quality of infrastructure like barriers and levees influences flood resilience.
**Population density and education** (Roldán-Valcarce et al. 2023)	Social	High population density and education levels affect preparedness and response.
**Sanitation and poverty** (Ajtai et al. 2023; Azmeri and Satria 2021)	Social	Sanitation access and poverty limit resources for recovery and public health during floods.
**Urban planning and investment in flood control** (Eini et al. 2020)	Economic	City planning and investment in flood control determine vulnerability and mitigation capacity.

Note: Please see the full reference list of the article, Mutia, E., Azmeri, A., Yulianur, A., Achmad, A. & Meilianda, E., 2025, ‘Multifactor analysis of urban pluvial flooding using a comprehensive vulnerability index’, *Jàmbá: Journal of Disaster Risk Studies* 17(1), a1835. https://doi.org/10.4102/jamba.v17i1.1835, for more information.

## Conclusion

This study evaluated flood vulnerability in Langsa city using the FVI, identifying that low-lying areas with dense populations and inadequate drainage are the most at risk. The lack of green spaces and limited adaptive capacity further aggravate the situation, making flood mitigation an urgent necessity. The city’s flat terrain and high population density contribute significantly to heightened flood risks, with vulnerable social groups, particularly low-income residents, being the most affected.

To address these challenges, several strategies are recommended, including enhancing drainage infrastructure, optimising land-use planning, expanding access to sanitation services and implementing early warning systems. Community-based mitigation efforts, when aligned with strategic spatial planning, can significantly strengthen local resilience and reduce the impact of floods on at-risk populations. These measures are vital to reducing sensitivity to flooding and protecting the well-being of Langsa’s residents.

Future research should focus on improving flood prediction models that integrate climate change and urbanisation trends. Additionally, the adoption of real-time mapping technologies and collaborative efforts across sectors will be essential in building stronger, more sustainable urban flood mitigation systems.
